# Characterization of the complete chloroplast genome sequence of *Cardamine lyrata* Bunge(Brassicaceae)

**DOI:** 10.1080/23802359.2022.2079106

**Published:** 2022-06-02

**Authors:** Xinhan Xu, Xuan Yao, Caijuan Zhang, Pengguo Xia

**Affiliations:** a Key Laboratory of Plant Secondary Metabolism and Regulation of Zhejiang Province, College of Life Sciences and Medicine, Zhejiang Sci-Tech University, Hangzhou, China; b Hangzhou Sanyeqing Agricultural Science and Technology Co. Ltd., Hangzhou, China

**Keywords:** Brassicaceae, *Cardamine lyrata* Bunge, chloroplast genome, phylogenetic analysis

## Abstract

*Cardamine lyrata* Bunge 1833 grows near paddy fields, streams and shallow water. Its young stems and leaves can be eaten. It can also be used as medicine and has the effect of clearing away heat and dampness. The complete chloroplast genome sequence of the *C. lyrata* was determined and assembled. The complete genome is 155,170 bp in length, including a large single-copy region (LSC) of 84,270 bp, a small single-copy region (SSC) of 17,918 bp and two copies of inverted repeat (IR) regions of 26,491 bp. The overall GC content of *C. lyrata* is 36.2%. The genome of *C. lyrata* contains 131 genes, including 85 protein-coding genes (PCGs), 37 tRNAs, and 8 rRNAs. Phylogenetic analysis suggested that the ten species in *Cardamine* were clustered together into a single branch within the Brassicaceae family and *C. amariformis* is at the base of the tree and *C. lyrata* and *C. fallax* are sister groups of the inner clade.

*Cardamine*, one of the largest genus in Brassicaceae family. There are about 160 species of *Cardamine* in the world, and about 40 species in China (Li et al. 2021). *Cardamine lyrata* Bunge 1833, is a medicinal plant reported to have the effect of wind-dispelling and dampness (Hou et al. 2013). It mostly occurs at the edge of paddy field, stream and shallow water (Li and Zhong 1995). The rhizome is short and clustered with most fibrous roots. The stem is erect, unbranched, with furrowed edges on the surface. It usually produces slender and soft stolons from the leaf axil near the rhizome or the leaf axil at the lower part of the stem. In this study, we sequenced, assembled, and annotated the complete chloroplast genome of *C. lyrata*, which is helpful in promoting the development of *Cardamine*.

In this study, the materials of *C. lyrata* were from Zhenping County, Ankang City, Shaanxi Province (31°51′52.63″N, 109°34′11.4″E, and altitude 1206 m). A specimen was deposited at the Herbarium of Xi’an Botanical Garden (voucher number: *Xun Lulu* et al. *LB19895*, Lulu Xun, xunlulu20032006@126.com). The total genomic DNA of leaves was extracted by CTAB method (Doyle 1987) and sent to Majorbio (http://www.majorbio.com, China) for library construction and sequencing. Illumina NovaSeq 6000 platform was used for paired-end (PE) reads generation preparation with 2 × 150 bp PE reads. Libraries were size selected for 400 bp inserts. The raw sequencing data were Trimmed and filtered by Fastp software (Chen et al. 2018). The extracted DNA was deposited at Key Laboratory of Plant Secondary Metabolism and Regulation of Zhejiang Province, Zhejiang Sci-Tech University (http://sky.zstu.edu.cn) under the vocher number ZSTUX0102 (collected by Pengguo Xia and xpg_xpg@zstu.edu.cn). Using GetOrganelle v1.7.0 (Jin et al. 2020) assembled the entire chloroplast genome. Geneous Prime was used for gene annotation with reference to *C. fallax* (MZ043778). Genomic data were submitted to Genbank with serial number MZ846206.

The results showed that the complete chloroplast genome of was 155,170 bp, and the average GC content was 36.2%. The chloroplast genome consists of two reverse repeat regions (IR repeat) of 26,491 bp, a large single copy region (LSC) of 84,270 bp and a small single copy region (SSC) of 17,918 bp. We found that the complete chloroplast genome encoded 131 genes, including 85 protein coding genes, 37 tRNA genes and 8 rRNA genes.

In order to determine the phylogenetic position of *C. lyrata* in Brassicaceae, 17 Brassicaceae chloroplast genomes were downloaded from GenBank, and all protein coding gene sequences were compared with MAFFT (Nakamura et al. 2018). *Carica papaya* was used as the outgroup. The phylogeny was constructed by maximum likelihood (ML) method with IQTREE (Minh et al. 2020) software under the optimal model of TVM + F + R5 (Figure 1). The bootstrap value was 1000. The result of analysis showed that the *Cardamine* species formed a monophyletic clade within the Brassicaceae family and *C. lyrata* was the closest to *C. fallax.* Our results provide valuable data and shed light on the phylogenomic study of Brassicaceae.

**Figure 1.**
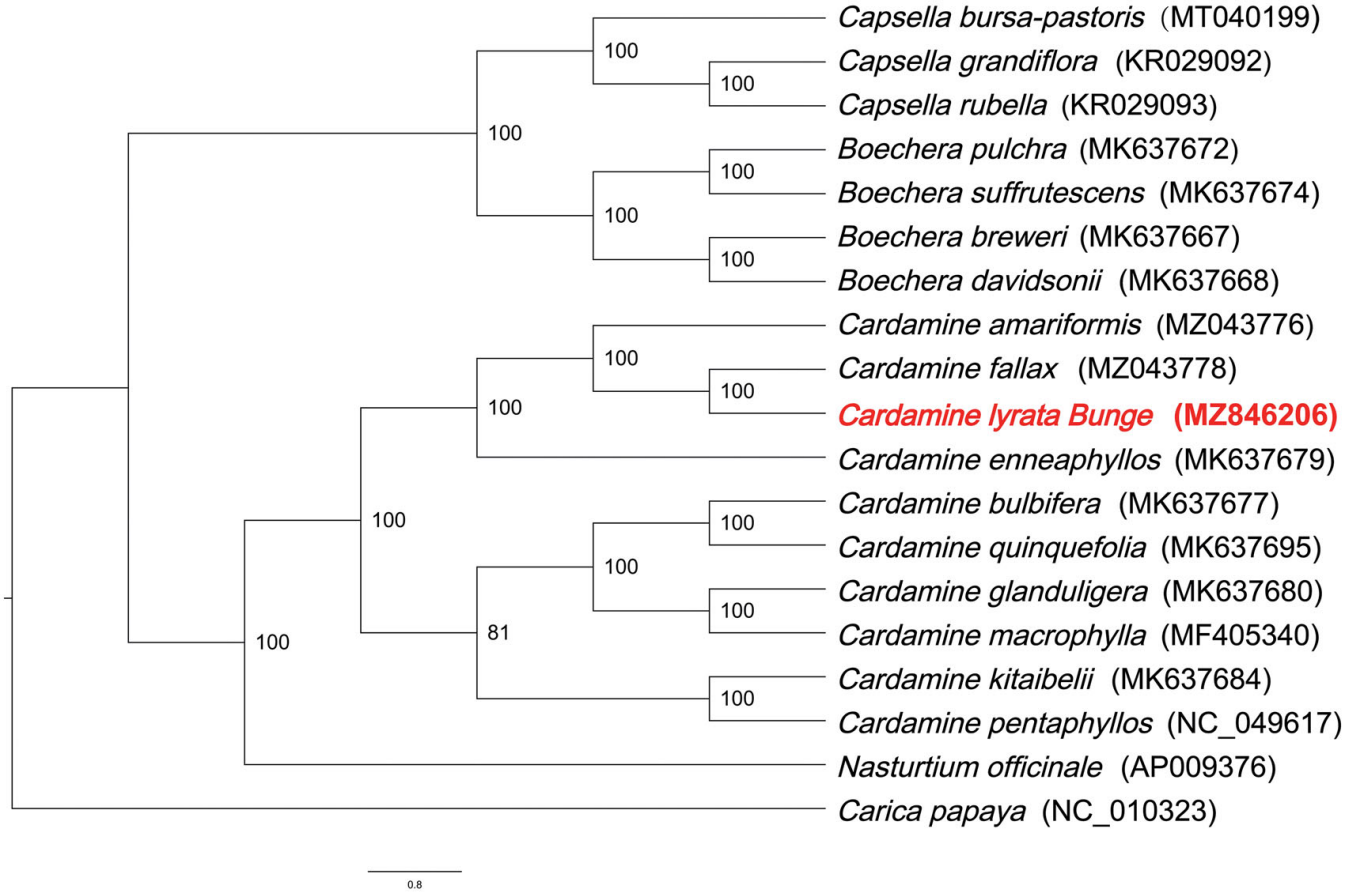
Maximum-likelihood tree of Cardamine base on complete chloroplast genomes, with Carica papaya as outgroup. The Cardamine lyrata Bunge is marked in red and Bootstrap support values (based on 1000 replicates) are shown next to the nodes.

## Ethical approval

Research and collection of plant material was conducted according to the guidelines provided by Herbarium of Xi’an Botanical Garden. Permission was granted by Herbarium of Xi’an Botanical Garden to carry out research on the species.

## Author contributions

P.X. conceived and designed this study. X. X. and C. Z. conducted analysis. X. X. and C. Z. contributed the analytical methods. X. Y. and C. Z wrote the manuscript. P.X. edited the manuscript. All authors have read and agreed to the published version of the manuscript.

## Data Availability

The data that support the findings of this study are openly available in NCBI (https://www.ncbi.nlm.nih.gov) GenBank with the accession number (MZ846206). The associated BioProject, SRA, and BioSample numbers are PRJNA756437, SRR15533325 and SAMN20865599, respectively.
